# Application of the Scale for the Assessment of Feeding Interaction (SVIA) to Children With Autism Spectrum Disorder

**DOI:** 10.3389/fpsyt.2019.00529

**Published:** 2019-07-24

**Authors:** Elena Catino, Giorgia Perroni, Michela Di Trani, Chiara Alfonsi, Flavia Chiarotti, Francesco Cardona

**Affiliations:** ^1^Azienda Universitaria Ospedaliera Policlinico Umberto 1, Rome, Italy; ^2^Department of Dynamic and Clinical Psychology, Sapienza University of Rome, Rome, Italy; ^3^Department of Human Neurosciences, Sapienza University of Rome, Rome, Italy; ^4^Center for Behavioral Sciences and Mental Health, Istituto Superiore di Sanità, Rome, Italy

**Keywords:** autism spectrum disorder, feeding disorder, mother-child co-regulation, direct observation, scale for the assessment of feeding interaction, brief autism mealtime behavior inventory

## Abstract

**Background and Objectives:** Feeding problems occur more frequently among children with Autism spectrum disorder (ASD). The aim of this study was to analyse eating difficulties of ASD children through the direct observation of the caregiver-child co-regulation system.

**Methods:** We compared 60 ASD children with a control group of 50 typically developing Italian children on the Scale for the Assessment of Feeding Interaction (SVIA). The Brief Autism Mealtime Behaviour Inventory (BAMBI) was used to define the presence of an eating disorder.

**Results:** The ASD group showed higher scores on all dimensions of the SVIA compared to the control group. The SVIA and the BAMBI showed significant correlations. In a second step, the ASD sample was divided into two subgroups, children with and without feeding difficulties. The comparison between the ASD subgroups with the control group on the SVIA scales showed significant differences on all dimensions. Finally, significant differences emerged between the two ASD subgroups in three SVIA dimensions.

**Conclusion:** These data suggest the importance of direct observation of feeding in the assessment of children with ASD. The SVIA seems to be able to point out some feeding difficulties in these subjects and to discriminate ASD with and without an eating disorder. Critical aspects of the application of SVIA to autistic children are discussed.

## Introduction

In early childhood, feeding is a precious moment of interaction (1). During feeding, children begin to recognise signals in their social (i.e., external) environment (vocalisations, glances, gestures, mimicking facial expressions) and internal signals of hunger/satiety (2–4). Studies on breastfeeding sequences reveal the intersubjective nature of nutrition: when an infant temporarily stops sucking, mothers use this break to talk to or touch him. This turn-taking—characterising early feeding interactions—is considered the first form of ‘dialogue’ between adult and infant (5).

In families with typically developing children, mealtimes are an opportunity to structure daily routines, which support social learning. Children spontaneously observe and imitate the actions of adults, who, in turn, adapt their actions and language to facilitate the child’s learning (6). This caregiver-child regulation system lays the foundation for future social skills (7–9), subsequent self-regulation skills (10) and is recognised as one of the predictors of developmental outcomes (1, 11, 12).

Autism spectrum disorders (ASDs) are a group of neurodevelopmental disorders that include a wide range of complex developmental disabilities. These include impairments in social interactions, diﬃculties with language and communication, repetitive and ritualised behaviours, restricted interests, behavioural inflexibility and impaired sensory processing (13). In particular, children with ASD tend be less accurate with identification of facial expressions (14) and they typically display problems with the quality and quantity of joint attention (15), besides presenting reduced eye contact and poor integration of eye contact with verbal and non-verbal communication (16). Children with ASD also can show a limited or immature motor repertoire and significant imitation deficits (17, 18). They often present characteristics including stereotyped movements, adherence to routines, resistance to change and intense preoccupations (13).

Early in life, in subjects with ASD, co-regulation processes between mother and child can be affected. Hirschler-Guttenberg et al. (19) analysed how children with ASD regulate both positive and negative emotions during free play with their mothers at home by micro-coding the behaviour of children. Observations indicated that pre-schoolers with ASD were less socially engaged and less compliant than typically developing peers. Similarly, a recent study by Guo et al. (20) found that mother-infant with ASD-dyads pass more time in mismatched emotion-engagement states (e.g., child negative/mother positive). Furthermore, children with ASD spent more time engaged exclusively with objects than children without ASD.

The caregiver–child relationship at mealtime has been studied mainly in the context of eating disorders or other feeding-related risk factors (21–27). To date, no studies have been conducted that examine the feeding relationships of children affected by ASD. The feeding of subjects with ASD has mainly been investigated through indirect observation methods (28). These studies have highlighted the strong association between feeding difficulties and autism. Feeding problems occur more frequently among children with ASD than typically developing children (29) or children with other disabilities (30). Food selectivity is the most common feeding disorder among the ASD population. These children show high rates of food refusal, a high frequency of singular food intake and a limited repertoire of accepted foods, with a tendency to maintain food restrictions over time (31, 32). Selectivity concerns sensory characteristics of foods (flavour, odour, colour, texture and temperature) (29, 33, 34). Additionally, children with ASD have been shown to have many eating rituals (32, 35, 36), as well as aggressive mealtime behaviours (37). Ritualistic and repetitive patterns of behaviour are commonly believed to contribute to food selectivity (32).

The new *Diagnostic and Statistical Manual of Mental Disorders*—5th edition (DSM-5) diagnostic criteria for avoidant/restrictive food intake disorder (ARFID), a childhood feeding disorder, encompasses some of the problems seen among children with ASD. The mechanisms of eating/feeding disorders in populations with ASD differ from those reported in children with feeding disorders without ASD; research has only begun to explore this difference. The meta-analysis by Sharp et al. (38) recommended including an assessment of feeding problems as part of routine medical evaluations, as well as developing assessment methods to identify empirically supported treatments for feeding problems in ASD.

The causes of eating/feeding problems in ASD are complex and include a combination of biological and environmental factors (32). Atypical feeding behaviours can impact the family feeding process and influence how families are forced to organise their mealtimes. For families of children with ASD, mealtimes can be overly focused on meal preparation and the sensory experiences of the child, which significantly limits opportunities for meaningful shared experiences (39). Therefore, the need exists to study ASD feeding disorders through direct observation methods, with a focus on the dyadic co-regulation system.

The aim of the present study was to analyse eating difficulties of children with ASD through direct observations of the caregiver-child co-regulation system. We compared ASD children with a typically developing Italian control group on the Scale for the Assessment of Feeding Interaction (SVIA), a measure of interactive behaviours that identifies relational models between parents and children during feeding sessions (Ammaniti M., Lucarelli L., Cimino S., D’Olimpio F. Scala di valutazione dell’interazione alimentare madre-bambino – SVIA; unpublished manuscript). We hypothesised that the clinical group would show higher scores on all the dimensions of the SVIA compared tothe control group. Moreover, in the ASD group, we analysed the relation between the SVIA and the Brief Autism Mealtime Behaviour Inventory (BAMBI), a self-reporting measure for the evaluation of mealtime behavioural problems in ASD children (40).

In a second step, we divided the ASD group into two subgroups, a subgroup with eating difficulties and a subgroup without eating difficulties, as assessed by the BAMBI. We hypothesised higher scores on the SVIA in both ASD groups with and without eating disorderscompared to the control group. Additionally, we hypothesised that ASD children with an eating disorder would show higher scores on the SVIA dimensions than ASD children without an eating disorder.

## Materials and Methods

### Participants

The participants included 60 families with children diagnosed with ASD or with a “high risk” of autism and 50 families of typically developing children.

The ASD sample was recruited from the Child Neuropsychiatry Unit of the Policlinico Umberto I, in Rome, Italy. The inclusion criteria in the study were: be affected by ASD following the DSM-5 criteria and be between 18 and 48 months of age. Exclusion criteria were: the presence of a known genetic disorder (e.g., Rett syndrome, Fragile X syndrome, Down syndrome), a medical disorder (e.g., epilepsy) or a history of environmental exposure (e.g., valproate, foetal alcohol syndrome, very low birth weight).

All parents provided written informed consent. The children were aged 20–44 months (mean ± SD = 32.27 ± 5.55). The sample included 49 males and 11 females. Diagnoses of ASD were based on the DSM-5 criteria (13). 38 children were assessed by modules 1 or 2 of the Autism Diagnostic Observation Schedule (ADOS) and 22 children were assessed by the toddler module (41). All ADOS-2 comparison scores were in the range of moderate or severe autism (module 1or 2) or of “high risk” of autism (toddler module). All children underwent a cognitive evaluation. In particular, 20 children were assessed through the Leiter International Performance Test-Revised (Leiter-R) (42); the average total IQ at the Leiter-R was 109.95 (SD = 14.45). The other 40 children, for whom a structured testing was not possible, were assessed through the Griffiths Mental Developmental Scale-Extend Revised (GMDS-ER) (43) and all had a development quotient below 24 months.

No child had first-degree relatives with ASD.

The control group was recruited from different kindergarten classes in Rome. The chief teachers of the schools were contacted in order to plan the recruitment of the participants in the study among their pupils with normal development and without relationship or communication problems. All parents provided written informed consent and stated that they never had access to child mental health services. Four recruited children were excluded from the study for the evidence of a slight language disorder. Ultimately, the control group included 34 males and 16 females, aged 18–43 months (mean ± SD = 29.88 ± 8.05).

### Procedures

The study was approved by the Ethics Committee of the Department of Dynamic and Clinical Psychology, Sapienza University of Rome, Italy. In the clinical group, all procedures were part of the assessment for ASD. The control group was assessed at home, after being recruited from the kindergarten classes. Parents signed a consent form prior to enrolment. Extensively trained clinicians conducted the assessments, which consisted of a videotaped parent-child interaction during mealtime.

### Measures

All mothers and their infants were observed using an Italian adaptation of the Feeding Scale – Scale for the Assessment of Feeding Interaction (Ammaniti M., Lucarelli L., Cimino S., D’Olimpio F. Scala di valutazione dell’interazione alimentare madre-bambino – SVIA unpublished manuscript), which can be applied to children between the ages of 1–36 months. This scale measures interactive behaviours and identifies relational models between parents and children during feeding sessions. Prior to taking part in the study, mothers of ASD children were instructed to bring foods that they would usually offer to their infants at home and to bring food for themselves, if they were accustomed to eating with their infants. The SVIA consists of 41 items distributed among 4 subscales (described below in detail): affective state of the mother, interactional conflict, food refusal behaviour of the child and affective state of the dyad. Each item received a score on the following Likert scale: 0 (*none*), 1 (*a little*), 2 (*pretty much*) and 3 (*very much*). A global rating was obtained for each subscale.

1) The *affective state of the mother* subscale (15 items, overall score from 0 to 45) refers to the possible difficulties of the caregiver in showing positive affect as well as the frequency and quality of expressed negative affect. It also evaluates the mother’s ability to interpret the child’s signals and facilitate reciprocal and empathic exchanges. Higher scores in this subscale indicate difficulties in expressing positive feelings and in correctly interpreting the infants’ needs.
2) The *interactional conflict* subscale (16 items, overall score from 0 to 48) evaluates both the presence and intensity of conflict within the dyad. This subscale score is high when, for example, the mother forces the child to eat or when she directs the meal according to her own emotions and intentions rather than following the communicative feedback of the child. High scores also result from the child showing behaviours of distress and avoidance in response to intrusiveness of the mother.
3) The *food refusal behaviours of the child* subscale (4 items, overall score from 0 to 12) includes items that only concern the child. This subscale explores the feeding patterns of the child, with high scores indicating food refusal, poor nutritional intake and difficulty in behavioural state regulation, such as irritability, hyper-excitability, being easily distracted, showing opposition and negativity.
4) The *affective state of the dyad* subscale (6 items, overall score from 0 to 18) evaluates the quality of affect in the mother–child interaction. A high score indicates negative involvement within the dyad, in which emotions of anger and hostility prevail. In this situation, the caregiver does not facilitate the child’s autonomous initiatives by, for example, exerting constant control during mealtime. As a consequence, the child is intensely reactive, showing distress.

Mother–infant interactions during feeding were recorded for at least 20 min. Two trained psychologists, blind to the infants’ diagnosis, rated the videotaped feeding interactions (Ammaniti M., Lucarelli L., Cimino S., D’Olimpio F. Scala di valutazione dell’interazione alimentare madre-bambino – SVIA; unpublished manuscript).

Children’s feeding difficulties were investigated with the BAMBI (40). The BAMBI is an 18-item parental report questionnaire that was designed to capture mealtime behaviours in children with ASD. The BAMBI is scored on a 1–5 Likert scale (1 indicating the behaviour “never” occurs at mealtime, 5 indicating the behaviour “always” occur at mealtime). Reversed scoring is used for four of the items rating positive mealtime behaviours. A total score (ranging from 18 to 90) is calculated from the sum of all items, with higher scores reflecting more mealtime behavioural problems. The BAMBI examines specific problem behaviours seen in populations with ASD. As such, it has strong potential for clinicians to assess feeding problems in children with ASD. The BAMBI was recently used in a large study on nutrition in 256 children with ASD, showing strong associations between BAMBI scores and repetitive and ritualistic behaviours, sensory features, as well as externalising and internalising behaviours (44). In the present study, we used the BAMBI cut-off score of 34 suggested by DeMand et al. (45), who investigated the psychometric properties of the BAMBI scale in a large, representative ASD sample (ages 2–11 years). These authors identified 4 factors: food selectivity, disruptive mealtime behaviours, food refusal and mealtime rigidity.

### Statistics

Quantitative data are summarised by means ± standard deviation (SD) and range. To compare ASD and control groups on the SVIA dimensions and to analyse the specific role of gender, a series of one-way analysis of covariances (ANCOVAs) were performed, including gender as a covariate. The Pearson’s linear correlation coefficient, r, was used to assess the association between the SVIA dimensions and the BAMBI total score. Finally, the ASD group was divided into two subgroups (based on the BAMBI cut-off score of 34) and one-way ANOVAs were performed to compare these two ASD groups with the control group. Specifically, Bonferroni *post hoc* analyses were applied to test differences among the three groups.

The number of subjects enrolled in the control and ASD groups (1. control, n = 50; 2. ASD, n = 60) allowed for detecting differences of small size (Cohen’s d = 0.54) at a two-tailed significance level = 0.05 and a power = 0.80. When performing pairwise comparisons between control subjects and ASD subjects with and without eating disorders, at a two-tailed significance level = 0.0167 (to take into account multiple comparisons by Bonferroni’s correction) and a power = 0.80, the numbers of subjects in the three subgroups (1. control, n = 50; 2.1. ASD with eating disorders, n = 13; 2.2. ASD without eating disorders, n = 44) are sufficient to detect differences of small/medium size (Cohen’s d = 1.03, 0.68, and 1.05, for comparison 1 vs 2.1, 1 vs 2.2, and 2.1 vs 2.2, respectively).

Statistical analyses were conducted using the Statistical Package for Social Science (SPSS) version 24.A *p*-value < 0.05 was considered significant for all analyses.

## Results

As shown in **Table 1**, ANCOVA analysis between the ASD group and control group on the SVIA dimensions showed higher scores on all the SVIA dimensions in the ASD group, than the control group. No effect of gender, included as a covariate, was found (*p* = 0.975).

**Table 1:** Analysis of covariances between ASD and control groups on SVIA dimensions.

**SVIA dimension**	ASD group n = 60		Control group n = 50		**F**	**p**
**Mean**	**SD**	**Mean**	**SD**		
Affective state of the mother	9.52	3.53	2.95	2.56	120.18	<0.001
Interactional conflict	8.79	4.04	4.17	2.45	49.92	<0.001
Food refusal	5.52	2.31	2.12	1.47	80.73	<0.001
Affective state of the dyad	3.06	1.96	1.29	1.29	29.64	<0.001

In the ASD group, Pearson’s correlation coefficients between the BAMBI score and the following dimensions of SVIA were statistically significant, even if the strength of the association was weak/moderate: Interactive conflict (r = 0.29, *p* = 0.03), Food refusal (r = 0.31, *p* = 0.02) and Affective state of the dyad (r = 0.27, *p* = 0.04). A feeble and non-statistically significant correlation was found between the BAMBI score and the Affective state of the mother dimension (r = 0.16, *p* = 0.246).

In a second step, the ASD sample was divided into two subgroups based on the BAMBI scores: 13 children with feeding difficulties (i.e., BAMBI score equal to or higher than the cut-off of 34) and 44 children without feeding difficulties (i.e., BAMBI score below the cut-off of 34). Three mothers did not fill out the questionnaire and their data were not included in the comparison. We compared these two subgroups with the control group on the SVIA scales and significant differences emerged on all dimensions. Specifically, *post hoc* analyses (Bonferroni) showed significant differences between both ASD subgroups, with and without an eating disorder, and the control group on all the SVIA dimensions (*p* < 0.001 for all).

Finally, significant differences emerged between the two ASD subgroups in three SVIA dimensions (Interactive conflict, Food refusal and Affective state of the dyad), but not in the Affective state of the mother dimension (**Table 2**).

**Table 2:** ANOVA between ASD subgroups with and without eating disorder on SVIA dimensions.

SVIA dimension	ASD group with eating disorder n = 13		ASD group without eating disorder n = 44		Control group n = 50		F	p
	Mean	SD	Mean	SD	Mean	SD		
Affective state of the mother	10.52	3.08	9.25	3.73	2.95	2.56	58.80	0.013
Interactional conflict	11.20	3.70	8.17	3.96	4.17	2.45	31.03	0.003
Food refusal	7.02	2.10	5.02	2.20	2.12	1.47	48.10	0.005
Affective state of the dyad	4.34	1.94	2.68	1.84	1.29	1.29	21.07	0.613

## Discussion

The aim of this study was to deepen our understanding of the patterns of food regulation in children with ASD, within the caregiver-child relationship. In particular, we evaluated the applicability of the SVIA in a group of children with ASD.

The comparison between groups on the SVIA showed higher scores on all the SVIA dimensions in the ASD group than the control group (no effect of gender, used as a covariate, emerged). These data were in line with the results of previous studies about the relation between autism and feeding disorders (29). Specifically, ASD children showed higher scores on the SVIA Food refusal dimension, compared to the control group; ASD children often got up and wandered without a specific purpose, likely as a result of the greater attention they paid to objects than to people. Furthermore, the presence of stereotyped behaviour led these children to physically isolate themselves, a behaviour which did not support food interactions.

Moreover, ASD children showed higher scores on the SVIA Affective state of the mother and Interactional conflict dimensions; the communicative deficits in ASD children affect the co-regulation processes between mother and child (19). Indeed, the caregivers of the clinical group showed more difficulties in expressing positive feelings and in correctly interpreting the children’s needs. In our study, ASD children showed reduced spontaneous initiation of interactions. Finally, ASD children showed higher scores on the SVIA Affective state of the dyad, as the clinical group had more difficulties in forming autonomies than the control group, possibly due to motor impairment. In these circumstances, mothers of ASD children were less likely to encourage engagement and attention towards the shared purpose of eating, than the mothers of the control group. As a consequence, the dyads of the clinical group showed more behaviours of distress, as well as negative involvement.

Regarding the relations between SVIA and BAMBI, in the ASD group, SVIA dimensions and the BAMBI score showed significant, though moderate correlations, except in the Affective state of the mother dimension. Several items of this subscale refer to the possible difficulties of the caregiver in showing positive affect, as well as the frequency and quality of expressed negative affect. Regarding the caregivers, these data could be explained as the effect of the parent’s mutual adaptation to the child’s communicative difficulties. We know from previous studies that children with ASD can send “unrecognisable signals.” Parents of our ASD children were prone to fail in this primary task, which could be related to the fact that the study was conducted during a diagnostic assessment phase, when the child’s problems are not yet well known. In other words, this result could be related to the effects of the child’s atypical communication on the dynamic characteristics of the co-regulation system, not to the eating disorder in ASD.

However, given the cross-sectional design of the study, we cannot rule out that the difficulties of the mother in regulating negative affect expression, as well as difficulties in expressing positive feelings and in correctly interpreting the infants’ needs could be related to individual characteristics of the mothers themselves.

Based on the increased probability of children with ASD developing an eating disorder (29), as well as the similarities between the DSM-5 criteria for ARFID and eating difficulties in children with ASD (13), we hypothesised that ASD children with eating disorders would show higher scores on the SVIA dimensions than ASD children without eating disorders. Our results partially confirmed these hypotheses, showing higher scores in the group of ASD children with eating disorders, than the control group, on the SVIA Affective state of the mother, Interactional conflict and Food refusal dimensions.

In fact, the data showed that the mothers of ASD children with eating disorders seemed to exhibit more intrusive interactions than mothers of ASD children without an eating disorder. The caregiver did not facilitate the child’s autonomous initiatives by exerting a constant control (“Mother waits for infant to initiate interactions”; “Mother distracts or allows infant to distract during feeding”). As a consequence, the mother-ASD child-dyads showed a high interactive conflict during mealtime and the levels of distress were higher than in the mother-non-ASD child-dyads.

The main limitations of this study include the small sample size, the use of a version of the BAMBI not validated in Italy for the assessment of eating disorders and the different contexts in which the observations of feeding interactions were conducted.

The main strength of the study is that the direct observation of feeding in the assessment of children with ASD was employed, but some critical reflection can be proposed about this application. The SVIA seems to be able to point out some feeding difficulties in these subjects and to discriminate ASD with and without eating disorder. The application of the SVIA also allowed for the evaluation of autistic children in an ecological context, enriching existing clinical information and providing applicable possibilities for improving relationships. In children under 30 months of age, the area of restricted and repetitive behaviours may be more evident during mealtimes. In particular, the child may exhibit more mannerisms triggered by contact with food and feeding routines.

Mealtimes promote regular interactive exchanges and create repeated social learning opportunities for children with ASD. In this context, children with ASD, like typically developing children, begin to experience the sharing of affection and reciprocal communication with their caregivers. The information obtained by SVIA in this study was shared with parents in order to support social reciprocity and communication exchanges.

At the same time, from a clinical point of view, the application of the SVIA highlighted some critical aspects of this instrument in a group of children with autism. In particular, the ASD children, with or without a feeding disorder, tended to have high scores in the areas of social interaction and emotional regulation of the SVIA, which cannot be explained as avoidance of the relationship or as an effect of the parent’s inability to respond appropriately to the child’s signals. The limited variability of facial expressions and the atypical eye contact of children with ASD (14, 16), that is recognised in many items of the SVIA, may have led to such pathological scores. These results are in line with studies on the peculiarity of expression and regulation of emotions in parent-child dyads with ASD (46). These data need to be interpreted in light of the neurodevelopmental and communicative deficits of children with ASD.

In conclusion, the identification of behavioural patterns regarding feeding has fundamental implications for early interventions. In the future, it will be important to adopt a multidimensional model in evaluating autistic children with food difficulties. Future models should include children’s biological, psychological and social maturation factors, considering the specific symptomatology/functioning of autism and the developmental patterns of the relationship between caregivers and children with ASD.

## Data Availability

The datasets generated for this study are available on request to the corresponding author.

## Ethics Statement

This study was carried out in accordance with the recommendations of Ethic Committee of the Department of Dynamic and Clinical Psychology, Sapienza University of Rome, Italy, with written informed consent from all parents. All parents gave written informed consent in accordance with the Declaration of Helsinki. The protocol was approved by the Ethic Committee of the Department of Dynamic and Clinical Psychology, Sapienza University of Rome, Italy.

## Conflict of Interest Statement

The authors declare that the research was conducted in the absence of any commercial or financial relationships that could be construed as a potential conflict of interest.

## Author Contributions

EC and MT contributed to the conception and design of the study, performed the statistical analysis, and wrote the first draft of the manuscript. GP and CA assessed the subjects and organized the database. FCh revised the statistical analysis. FCa wrote the sections of the manuscript and revised the manuscript. All authors contributed to manuscript revision and read and approved the submitted version.
